# Anti-inflammatory role of GM1 and other gangliosides on microglia

**DOI:** 10.1186/s12974-021-02374-x

**Published:** 2022-01-06

**Authors:** Danny Galleguillos, Qian Wang, Noam Steinberg, Asifa Zaidi, Gaurav Shrivastava, Kamaldeep Dhami, Gour C. Daskhan, Edward N. Schmidt, Zoë Dworsky-Fried, Fabrizio Giuliani, Matthew Churchward, Christopher Power, Kathryn Todd, Anna Taylor, Matthew S. Macauley, Simonetta Sipione

**Affiliations:** 1 Department of Pharmacology, University of Alberta, 9-21 Medical Sciences Building, Edmonton, AB T6G 2H7 Canada; 2 Department of Medicine, University of Alberta, Edmonton, AB Canada; 3 Department of Psychiatry, University of Alberta, Edmonton, AB Canada; 4 Department of Chemistry, University of Alberta, Edmonton, AB Canada; 5 Neuroscience and Mental Health Institute, University of Alberta, Edmonton, AB Canada; 6 Department of Medical Microbiology and Immunology, University of Alberta, Edmonton, AB Canada

**Keywords:** Gangliosides, GM1, GENZ-123346, L–*t*-PDMP, Liposomes, Microglia, BV2 cells, LPS, Inflammation

## Abstract

**Background:**

Gangliosides are glycosphingolipids highly enriched in the brain, with important roles in cell signaling, cell-to-cell communication, and immunomodulation. Genetic defects in the ganglioside biosynthetic pathway result in severe neurodegenerative diseases, while a partial decrease in the levels of specific gangliosides was reported in Parkinson’s disease and Huntington’s disease. In models of both diseases and other conditions, administration of GM1—one of the most abundant gangliosides in the brain—provides neuroprotection. Most studies have focused on the direct neuroprotective effects of gangliosides on neurons, but their role in other brain cells, in particular microglia, is not known. In this study we investigated the effects of exogenous ganglioside administration and modulation of endogenous ganglioside levels on the response of microglia to inflammatory stimuli, which often contributes to initiation or exacerbation of neurodegeneration.

**Methods:**

In vitro studies were performed using BV2 cells, mouse, rat, and human primary microglia cultures. Modulation of microglial ganglioside levels was achieved by administration of exogenous gangliosides, or by treatment with GENZ-123346 and L–*t*-PDMP, an inhibitor and an activator of glycolipid biosynthesis, respectively. Response of microglia to inflammatory stimuli (LPS, IL-1β, phagocytosis of latex beads) was measured by analysis of gene expression and/or secretion of pro-inflammatory cytokines. The effects of GM1 administration on microglia activation were also assessed in vivo in C57Bl/6 mice, following intraperitoneal injection of LPS.

**Results:**

GM1 decreased inflammatory microglia responses in vitro and in vivo, even when administered after microglia activation. These anti-inflammatory effects depended on the presence of the sialic acid residue in the GM1 glycan headgroup and the presence of a lipid tail. Other gangliosides shared similar anti-inflammatory effects in in vitro models, including GD3, GD1a, GD1b, and GT1b. Conversely, GM3 and GQ1b displayed pro-inflammatory activity. The anti-inflammatory effects of GM1 and other gangliosides were partially reproduced by increasing endogenous ganglioside levels with L–*t*-PDMP, whereas inhibition of glycolipid biosynthesis exacerbated microglial activation in response to LPS stimulation.

**Conclusions:**

Our data suggest that gangliosides are important modulators of microglia inflammatory responses and reveal that administration of GM1 and other complex gangliosides exerts anti-inflammatory effects on microglia that could be exploited therapeutically.

**Supplementary Information:**

The online version contains supplementary material available at 10.1186/s12974-021-02374-x.

## Background

Microglia, the myeloid cells of the central nervous system (CNS), play important homeostatic roles in health and disease. In the healthy adult CNS, microglia exert modulatory and housekeeping functions [1–3] that span from synapse remodeling and maturation to secretion of neurotrophic factors [4, 5] and regulation of the pool of neuronal precursors [6]. When signs of damage or infection are detected, microglia mount a highly regulated response that is generally defined as “microglia activation” and is tailored to the elimination of noxious triggers and pathogens and to the repair of tissue damage [7]. Inherent to microglia function in the CNS is their involvement in essentially all types of neurological and neurodegenerative conditions [8]. In many of these conditions, including Alzheimer’s disease (AD) [9], Huntington’s disease (HD) [10, 11], and Parkinson’s disease (PD) [12, 13], among others, a maladaptive increase in microglia inflammatory responses contributes to disease onset and/or progression [14–16].

Glycans play a major role in the regulation of immune cell functions [17]. The glycome of the CNS is predominantly composed of glycolipids, and more specifically gangliosides [18], in stark contrast to peripheral systems, where most glycans are carried by glycoproteins [19]. Gangliosides are glycosphingolipids made of a glycan headgroup containing one or more sialic acid residues attached through glycosidic linkage to a hydrophobic ceramide tail that anchors the gangliosides to the plasma membrane. The ganglioside headgroup can engage in *cis* interactions with proteins or other glycans present on the same membrane, as well as trans interactions with molecules on other cells and in the extracellular space, which result in modulation of cell signaling and cell-to-cell communication [20, 21]. The importance of gangliosides for brain health is highlighted by the fact that loss-of-function mutations that affect their synthesis cause neurodegeneration in humans and mice [21, 22]. A decrease in ganglioside levels as well as changes in the relative abundance of specific gangliosides also occur in ageing [23–26] and in common neurodegenerative conditions, including HD [27, 28], PD [29, 30] and AD [31, 32]. Therapeutic administration of one of the most abundant brain gangliosides, GM1, provides neuroprotection in models of neuronal injury and neurodegeneration [33–37] and in genetic models of HD [38, 39].

The mechanisms underlying the neuroprotective effects of endogenously synthesized and therapeutically administered gangliosides are only partially understood. Past studies have mainly focused on their effects in neurons, while their role in other brain cells remains largely unexplored or controversial. The few in vivo studies that have investigated the effects of lack of gangliosides or of exogenous ganglioside administration on microglia activation and neuroinflammation are often difficult to interpret, due to concomitant confounding effects in neuronal cells [40–42]. It is also unknown whether changes in endogenous ganglioside levels as observed in ageing and disease, or administration of exogenous gangliosides, affect microglia activation and neuroinflammation. Therefore, studies on isolated microglia are crucial to determine whether gangliosides play a modulatory role in microglia activation and to elucidate the neuroprotective effects of therapeutically administered gangliosides.

To address these questions, we used two complementary experimental paradigms: (1) administration of exogenous GM1 and other major gangliosides to determine the potential effects of therapeutically administered gangliosides on microglia and neuroinflammation; and (2) modulation of endogenous microglial ganglioside levels using a pharmacological activator and an inhibitor of glycolipid synthesis. The latter approach was used to mimic the partial decrease in ganglioside levels that has been observed in neurodegenerative conditions [27–29]. We demonstrate that administration of exogenous GM1 curtails the inflammatory response induced in microglia by stimuli such as LPS, IL-1β, or engulfment of latex beads. These anti-inflammatory effects depend on (1) the presence of the sialic acid residue in the glycan headgroup of GM1 and (2) the presence of a lipid tail. We further show that several other gangliosides, but not all, share anti-inflammatory properties with GM1. In line with the effects of exogenous gangliosides, increasing endogenous ganglioside synthesis decreases microglia inflammatory responses, while decreasing microglia ganglioside levels leads to a heightened response to LPS. Altogether, our results suggest that gangliosides play an important role in microglia activation and that modulation of their levels in microglia can offer new therapeutic avenues in neurodegenerative and neuroinflammatory conditions.

## Methods

### Animals and human cell cultures

C57BL6J mice were obtained from The Jackson Laboratory. All procedures on animals were approved by the University of Alberta Animal Care and Use Committee (AUP00000336) and were in accordance with the guidelines of the Canadian Council on Animal Care.

Human primary microglia cultures were prepared from fetal tissue obtained from 15 to 20 week-electively terminated healthy pregnancies with written informed consent of the donors (Pro000027660), as approved by the University of Alberta Human Research Ethics Board (Biomedical).

### Chemicals and reagents

Ganglioside GM1 (purified from porcine brain) was obtained from TRB Chemedica Inc. (Switzerland) and resuspended in cell culture grade D-PBS. Gangliosides GM3 and GD3 were obtained from Avanti Polar Lipids. GM2, GD2 and GQ1b were obtained from Cayman Chemical. GD1b, GD1a, GT1b, asialoGM1 (GA1) and GM1 pentasaccharide were purchased from Enzo Life Sciences. All gangliosides were > 98% pure according to manufacturers’ information. The truncated GM1-azide (tGM1) was kindly donated by Dr. David Bundle (University of Alberta). Lipopolysaccharide (LPS, serotype O55:B5, gamma-irradiated) was purchased from Sigma (Sigma L6529), recombinant mouse IL-1β was purchased from Cedarlane (Cedarlane CLCYT273), L-*threo*-1-phenyl-2-decanoylamino-3-morpholino-1-propanol·HCl (L–*t*-PDMP) was from Matreya LLC (Matreya #1749), N-[(1R,2R)-1-(2,3-Dihydrobenzo[b][1,4]dioxin-6-yl)-1-hydroxy-3-(pyrrolidin-1-yl)propan 2-yl] nonanamide (GENZ-123346) was obtained from Toronto Research Chemicals (TRC G363450) and solubilized in DMSO. 1,2-distearoyl-sn-glycero-3-phosphocholine (DSPC), cholesterol and 1,2-distearoyl-sn-glycero-3-phosphoethanolamine-N-[methoxy(polyethylene glycol)-2000] (ammonium salt) (PEG_45_-DSPE) were purchased from Avanti Polar Lipids. All other reagents were purchased from Sigma unless otherwise stated.

### Preparation of ganglioside-containing liposomes

Lipid stock solutions were prepared by dissolving an appropriate amount of each lipid in chloroform to reach the desired concentration [8 mg/ml DSPC, 4 mg/ml cholesterol and 0.1 mg/ml PEG_45_-DSPE (MW 2000)]. An appropriate volume of each lipid stock was transferred to a glass test-tube and the chloroform was evaporated under a gentle stream of nitrogen to produce a thin lipid film. Once all the chloroform was removed, 100 µl of DMSO was added to each test-tube. Individual gangliosides (GM1, GA1, GM3 and GM1-DSPE) dissolved in DMSO were then added at the appropriate concentrations to the glass tube and the lipid mixture was frozen at – 80 °C. DMSO was removed from the preparation by lyophilization O/N and the dried lipid mix was stored at – 80 °C until further processing. 1 ml of PBS pH 7.4 buffer was added to the lipids and samples were sonicated for 1 min followed by 5 min rest, for a total of 5 sonication cycles (approximately 30 min total). Liposomes were first extruded through a 400 nm filter and then through a 100 nm filter using an Avanti Mini-Extruded. The size of the liposome particles was determined via dynamic light scattering and found to be 110 nm ± 20 nm.

### Synthesis of GM1-DSPE

Truncated GM1-azide (tGM1; Additional file 1: Fig. S4I) was dissolved in a solution of pyridine, Et_3_N and water and cooled to 0 °C in an ice bath. H_2_S gas was bubbled through the solution for about 15 min. The reaction mixture was capped and stirred O/N at room temperature. The product was concentrated to near dryness and the pyridine was co-evaporated off with water. The residue was suspended in MeOH and centrifuged to remove off the white solids. The solvent was removed under vacuum and the crude product was dissolved in water. The crude product was loaded into the C-18 Sep-Pak cartridge, previously pre-equilibrated by eluting with 1% DIPEA in MeOH to MeOH/H_2_O (1:9 to pure water), using 0.1% DIPEA in H_2_O, and then purified using H_2_O to MeOH/H_2_O (~ 1:9 to 3:7, v/v, ~ 4–5 ml) as an eluent to obtain GM1-amine (Additional file 1: Fig. S4II). To synthesise the GM1-DSPE conjugate, an amine coupling was performed by dissolving GM1-amine in anhydrous DMF in a dried vial and combining it with NHS-activated-DSPE and Et_3_N to adjust pH of the solution to ~ 7.5–8.0 in the same solvent, at room temperature. The reaction mixture was stirred at the room temperature for 18 h. The solvent was removed under reduced pressure and the remaining crude product was loaded onto Sephadex G-100 gel filtration column using H_2_O to obtain the conjugate GM1-DSPE conjugate (Additional file 1: Fig. S4III) with 85% yield. Analysis of the 1H spectrum of GM1-DSPE confirmed formation of the desired conjugate with 60% coupling efficiency.

### Primary cultures and cell lines

Primary mixed glial cultures from P0.5–P1.5 mice (C57BL/6J or FVB/NJ strain) and rats (Sprague Dawley) were prepared following the method described by [43]. In brief, cerebral cortices were enzymatically and mechanically dissociated and cells were seeded in DMEM/F12 medium supplemented with 10% FBS, 100 units/ml penicillin, 100 µg/ml streptomycin, 1 mM sodium pyruvate and 50 µM β-mercaptoethanol. The growth medium was replaced every 4 days. On day 14, the medium was removed, and cultures were trypsinized to remove the monolayer of astrocytes leaving adherent microglia attached to the bottom of the culture dish. Isolated microglia were left to rest for 24 h in DMEM/F12 without FBS (supplemented with 1 mM sodium pyruvate and 50 µM β-mercaptoethanol) before any experimental treatment. Human fetal microglia were prepared as previously reported [44], from human fetal tissue obtained from 15 to 20 week-electively terminated healthy pregnancies. Briefly, fetal brain tissue was dissected, meninges were removed, and a single-cell suspension was prepared by enzymatic digestion followed by passage through a 70-μm cell strainer. Cells were plated in T-75 flasks and maintained in MEM supplemented with 10% FBS, 2 mM L-glutamine, 1 mM sodium pyruvate, MEM nonessential amino acids, 0.1% dextrose, 100 U/ml penicillin, 100 μg/ml streptomycin, 0.5 μg/ml amphotericin B, and 20 μg/ml gentamicin. Mixed cultures were maintained for 2 weeks. Weakly adhering microglia were recovered by gently rocking the mixed cultures for 20 min, followed by cell decanting, washing and plating onto 96 well plates (50,000 cells/well). Isolated microglia were allowed to rest for 3 days before performing experiments. BV-2 cells [45] (kindly donated by Dr. Jack Jhamandas, University of Alberta) were grown in RPMI-1640 supplemented with 10% FBS, 1 mM sodium pyruvate, 2 mM L-glutamine and 50 µM β-mercaptoethanol. All cells were maintained at 37 °C in 5% CO_2_.

### Cell treatments

GM1 was applied to microglia concomitantly with LPS or after washing off LPS from the cells, as described below. In the former case, after microglia isolation and resting in medium without FBS for 24 h, the medium was replaced with DMEM/F12 supplemented with 1 mM sodium pyruvate and 50 µM β-mercaptoethanol, with or without 50 µM GM1. After 1 h (for BV2 cells) or 2 h pre-incubation (for primary microglia), LPS was added directly to the medium at a final concentration of 100 ng/ml for 24 h. Treatment with IL-1β (5 ng/ml) was performed using a similar protocol. In a second set of experiments, after microglia isolation and resting as described above, LPS (100 ng/ml) was added to the cultures for 3 h. Cells were then washed once with HBSS containing Ca^2+^ and Mg^2+^ (HBSS^++^) and 0.1% essentially fatty acid-free BSA, once with HBSS^++^ and twice more with DMEM/F12. Immediately after washing, cells were cultured for 7–8 h in GM1, GM1ps, tGM1, GD3, GM2, GD2, GD1b, GD1a, GT1b or GQ1b (all 50 µM in DMEM/F12 supplemented with 1 mM sodium pyruvate and 50 µM β-mercaptoethanol). Except for the more soluble GMps and tGM1, at the concentration used these gangliosides are expected to form micelles in aqueous solutions. Liposome-bound GM1, GM1-DSPE, GA1 and GM3 were used at 200 µM (liposomal concentration, carrying a ganglioside mass equivalent to 6 µM of ganglioside in solution). For experiments with L–*t*-PDMP and GENZ-123346, BV-2 cells and primary microglia were treated for a total of 72 h with the compound in medium containing 5% FBS. After 48 h of treatment, half of the medium was removed and fresh medium with L–*t*-PDMP or GENZ-123346 was added. Subsequent incubation with LPS was performed in serum-free medium.

### Phagocytosis of latex beads

To evaluate microglia activation and phagocytic activity towards latex beads, the Phagocytosis Assay Kit (IgG FITC) (Cayman Chemical 500290) was used according to the manufacturer’s instruction. Briefly, BV-2 cells were incubated with 1 µm FITC-beads for 2 h at 37 °C, unbound beads were washed away with cold PBS, and trypan-blue was added for 2 min at room temperature to quench the fluorescence of any remaining beads on the surface of microglia. Phagocytosis of latex beads by BV-2 cells was quantified by flow cytometry in the Flow Cytometry Core Facility of the Faculty of Medicine & Dentistry at the University of Alberta and analyzed with FlowJo software.

### Intraperitoneal LPS and intraventricular administration of GM1 in mice

LPS (5 mg/kg in sterile saline solution) was injected intraperitoneally. After 3 days, a mini-osmotic pump (Alzet model 1002) filled with 3.6 mM GM1 in artificial cerebro-spinal fluid (aCSF, Harvard Apparatus, Holliston, MA) was implanted under the mouse dorsal skin and connected to a cannula inserted into the mouse brain third ventricle, as previously described [38, 39]. The pump infused GM1 into the brain ventricle at a flow rate of 0.25 μL/h for 3 days. Control animals were infused with aCSF. Slow-release buprenorphine (0.5 mg/kg) was administered to all animals during sedation to alleviate post-surgical pain.

### Analysis of brain microglia numbers and morphology

The brains were extracted from euthanized animals and immersed in freshly prepared fixative (4% PFA dissolved in 0.2 M sodium phosphate buffer) for 48 h at 4 °C, followed by immersion in 30% sucrose until tissue sank. Tissues were embedded in OCT, frozen on dry ice and stored at − 80 °C. The frozen brain section (25 µm-thick) were cut using a cryostat and mounted onto glass slides. Two sections per brain between + 0.18 and − 1.15 from bregma were incubated with rabbit anti-Iba-1 antibodies (1:500, Wako) O/N at 4 °C. The next day, secondary immunolabeling was performed using donkey anti-rabbit IgG Alexa Fluor 488 (1:200, Invitrogen-Life Technologies). Tissue sections were counter-stained with ProLong Gold mounting media containing DAPI (Invitrogen) to visualize cellular nuclei. For each slide, 4 photomicrographs were randomly taken in the cortex and 4 in the striatum region using an Axio Imager M2 microscope (Zeiss) with a 20X objective. Morphology and the number of Iba-1^+^ cells were analyzed using MetaXpress software (Molecular Devices). Images were imported into MetaXpress for analysis with the Neurite Outgrowth Application Module (Molecular Devices). Iba-1^+^ cells were identified according to the following cell body parameters: *max. width* (25 pixels); *signal intensity above local background* (4500 Gy levels); *min. area* (350 pixels). Iba-1^+^ cells meeting these criteria were included in the analysis and the following parameters were measured: total cell number, cell body area (μm^2^), number of processes and number of branching points.

### Immunoblotting

BV-2 cells and primary microglia were lysed in ice-cold 20 mM Tris, pH 7.4, containing 1% Igepal CA-630, 1 mM EDTA, 1 mM EGTA, 1X cOmplete protease inhibitor and PhosStop phosphatase inhibitor cocktails (Roche) and 50 µM MG-132. Mouse brain tissues were lysed in ice-cold 50 mM Tris, pH 7.5, containing 150 mM NaCl, 1 mM EDTA, 1X cOmplete protease inhibitor and PhosStop phosphatase inhibitor cocktails (Roche). For immunoblotting, 20 μg of proteins from BV-2 cells were separated by 10% SDS-PAGE, or 50 µg of brain lysate were separated by 15% SDS-PAGE and transferred to Immobilon-FL membranes (Millipore). For dot-blot analysis of gangliosides, 2 μg of proteins were spotted onto nitrocellulose membranes (Bio-Rad, pore size 0.45 µm) using a dot-blotting apparatus (Bio-Rad) according to manufacturer’s instruction. After blocking in Odyssey blocking buffer (LI-COR) for 1 h, membranes were incubated overnight at 4 °C with the following primary antibodies: rabbit anti-IKKα (Cell Signaling 2682; 1:1000), rabbit anti-phospho-IKKα/β (Ser180/Ser181) (Cell Signaling 2681; 1:1000), rabbit anti-p38 MAPK (Cell Signaling 9212; 1:1000), rabbit anti-phospho-p38 MAPK (Thr180/Tyr182) (Cell Signaling 9211; 1:1000), goat anti-Iba-1 (Novus Biologicals NB100-1028; 1:500), mouse anti-α-tubulin (Sigma T5168; 1:5000), rabbit anti-GM1 (Calbiochem 345757: 1:1000), mouse anti-GD1a (Millipore MAB5606Z; 1:1000), mouse anti-GD1b (DSHB GD1b-1 1:200), mouse anti-GT1b (Millipore MAB5608; 1:500) or cholera toxin subunit B-Alexa647 (Invitrogen C34778, 1 µg/ml). Incubation with the appropriate IRDye secondary antibodies (LI-COR, 1:10,000) was performed for 1 h at room temperature. Infrared signals were acquired and quantified using an Odyssey Imaging System (LI-COR) instrument.

### RNA extraction and qPCR analysis

Primary microglia or BV-2 cells were collected in RLT buffer (QIAGEN). RNA was isolated using RNEasy Micro (for primary microglia) or Mini Kit (for BV2 cells). cDNA was synthesized from 200 to 500 ng of RNA and reverse transcribed using Oligo dT primers and SuperScript II (Invitrogen). qPCR was carried out using PowerUp SYBR Green Master Mix (Applied Biosystems) in a StepOne Plus instrument (Applied Biosystems). Unless otherwise indicated, gene expression was normalized over the geometric mean of the expression of 3 reference genes: Atp5b, Cyclophilin A and Rplp0 (Normalization Index), according to [46].

### Analysis of cytokines

Cytokines released by primary microglia in the culture medium were quantified by ELISA using the following commercial kits according to the manufacturer’s instruction: Mouse TNF alpha Uncoated ELISA kit (88-7324-22, Invitrogen), Rat IL-6 DuoSet ELISA (DY506) and Rat IL-1β DuoSet ELISA (DY501) (R&D Systems), and Human IL-1β DuoSet ELISA (DY201, R&D Systems). Cytokines and growth factors levels were normalized to total protein content in the corresponding cell lysates. For the quantification of cytokines in brain homogenates (TNF, IL-6), aliquots of brain homogenates containing equivalent amounts of proteins were analysed using a Luminex platform (Thermo Fisher Scientific) by Eve Technologies Corporation (Calgary, AB, Canada).

### Statistical analysis

Two-tailed *t*-test analysis, one-way ANOVA corrected for multiple comparisons (Sidak’s post-test) or two-way ANOVA corrected for multiple comparisons (Tukey’s post-test) were performed as indicated in the figure legends, using GraphPad Prism 9. In all figure legends, N refers to the number of independent experiments.

## Results

### Administration of exogenous GM1 decreases microglia activation following pro-inflammatory stimulation with LPS

**Fig. 1:**
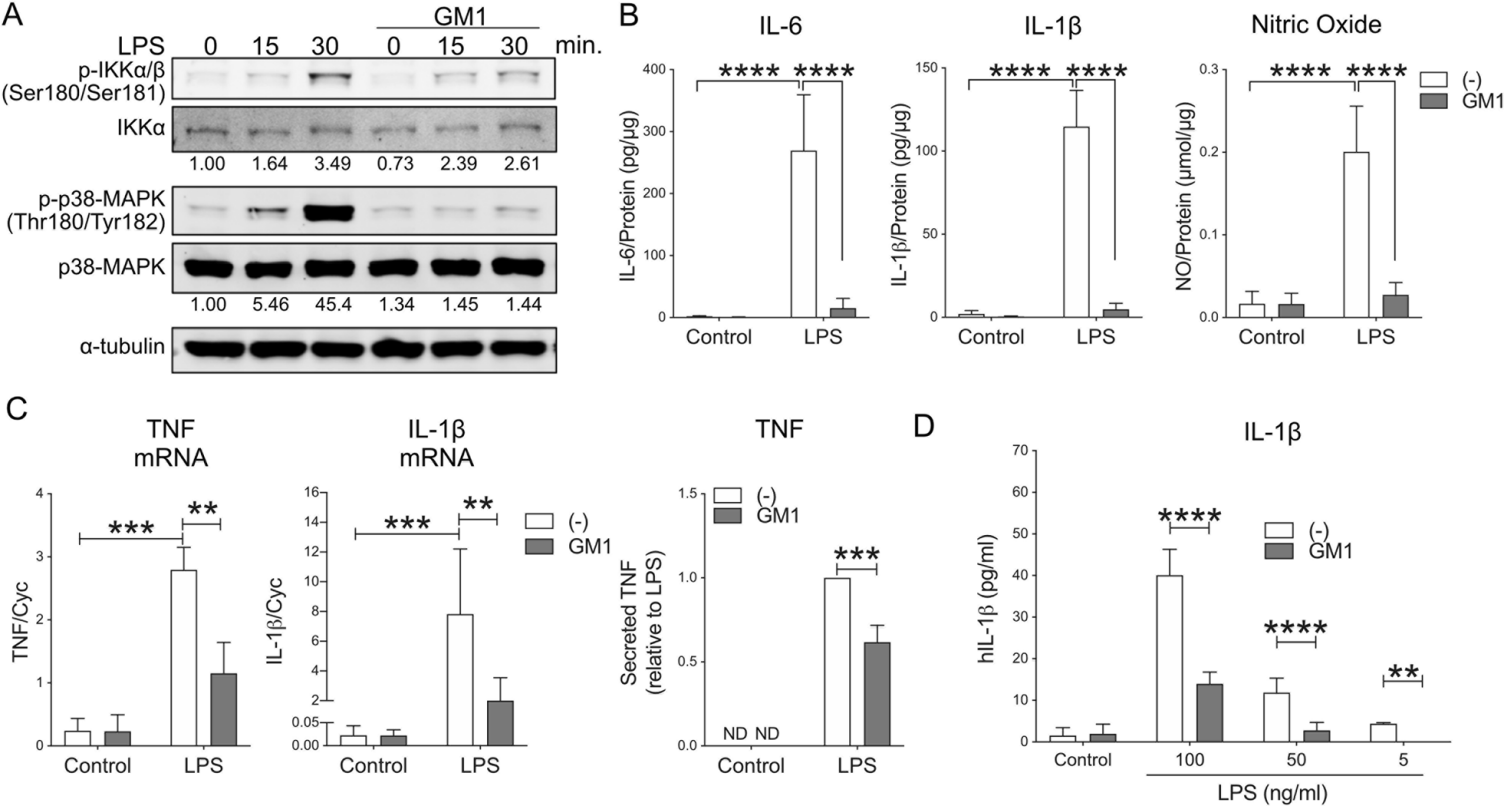
Administration of GM1 before or after microglia stimulation with LPS curtails pro-inflammatory microglia activation. **A** BV2 microglial cells were pre-incubated with GM1 (50 µM) or vehicle for 1 h prior to stimulation with LPS (100 ng/ml). Representative immunoblots (of 3 independent experiments) show decreased levels of phospho-IKKα/β and phospho-p38 MAPK following stimulation with LPS in cells pre-treated with GM1. The numbers under the immunoblots are densitometric measurements for phospho-IKKα/β and phospho-p38 MAPK normalized over total protein, and show fold-change over unstimulated controls. **B** Rat primary microglia were pre-incubated with GM1 for 2 h followed by stimulation with LPS (100 ng/ml, 24 h) prior to measuring IL-6, IL-1β and nitric oxide (NO) released in the medium (*N* = 3 independent experiments). **C** Mouse primary microglia were stimulated for 3 h with LPS (100 ng/ml), washed and further incubated with GM1 (50 µM) for 8 h. Expression of TNF and IL-1β mRNA and TNF secreted in the medium were significantly decreased in GM1-treated cells (*N* = 3–5). **D** Human fetal microglia were treated as in **C**. GM1 treatment decreased IL-1β secretion into the medium (*N* = 3)

To determine the specific effects of exogenous GM1 administration on microglia in inflammatory conditions, we initially used BV2 microglia cells, an extensively used and easy to grow microglia model that recapitulates many primary microglia behaviors in response to inflammatory factors [45, 47]. BV2 cells were pre-incubated with GM1 (50 µM) for 1 h and then stimulated with LPS (*E. coli* serotype O55:B5, 100 ng/ml) to activate the Toll-like receptor 4 (TLR-4), a major pattern recognition receptor that is also activated by endogenous danger-associated molecular patterns released in stress and neurodegenerative conditions [48–50]. The concentration of GM1 was chosen based on initial dose–response experiments (Additional file 1: Fig. S1A) and previous studies that showed neuroprotective properties of GM1 at a concentration of 50 µM [27]. At this concentration GM1 forms micelles in an aqueous solution [51]. As expected, LPS stimulation induced activation of the NFkB and the MAPK pathways, as shown by phosphorylation of IKK and p38 MAPK, respectively (Fig. 1A). In cells pre-treated with GM1, this response was significantly attenuated (Fig. 1A) and correlated with a dramatic decrease in the downstream expression of NFkB pro-inflammatory target genes, including TNF and IL-1β (Additional file 1: Fig. S1B). We confirmed these results in primary cultures of mouse and rat microglia, where the administration of GM1 2 h prior to a challenge with LPS blocked the transcription of NFkB target genes, including IL-1β, TNF, and IκBα (Additional file 1: Fig. S1C), and the release of pro-inflammatory cytokines IL-6, IL-1β, and NO in the culture medium (Fig. 1B). The effects of GM1 on microglia activation were not due to a decrease in cell viability (Additional file 1: Fig. S2A) or to a decrease in cell surface expression of TLR-4 (Additional file 1: Fig. S2C), although cells treated with GM1 for 24 h did show a decrease in total cellular TLR-4 compared to untreated cells (Additional file 1: Fig. S2D).

Next, we studied whether GM1 would dampen microglia inflammatory responses when administered *after* cell stimulation with LPS, as in most potential therapeutic settings, the ganglioside would likely be administered in the context of an already active inflammatory process. In cells pre-stimulated with LPS, GM1 treatment still decreased TNF and IL-1β gene expression (Fig. 1C), with an *IC*_50_ of 4.6 µM for TNF inhibition and 16.4 µM for IL-1β (Additional file 1: Fig. S1A) and decreased the amount of TNF released into the culture medium (Fig. 1C), without affecting cell viability (Additional file 1: Fig. S2B). Importantly, the anti-inflammatory effects of GM1 were reproduced in human fetal microglia, where treatment with the ganglioside after stimulation with LPS significantly decreased the secretion of IL-1β (Fig. 1D). Thus, GM1 administration attenuated microglial pro-inflammatory activation in all culture systems used in this study, not only when administered prior to LPS stimulation, but also when given to pre-activated microglia.

**Fig. 2:**
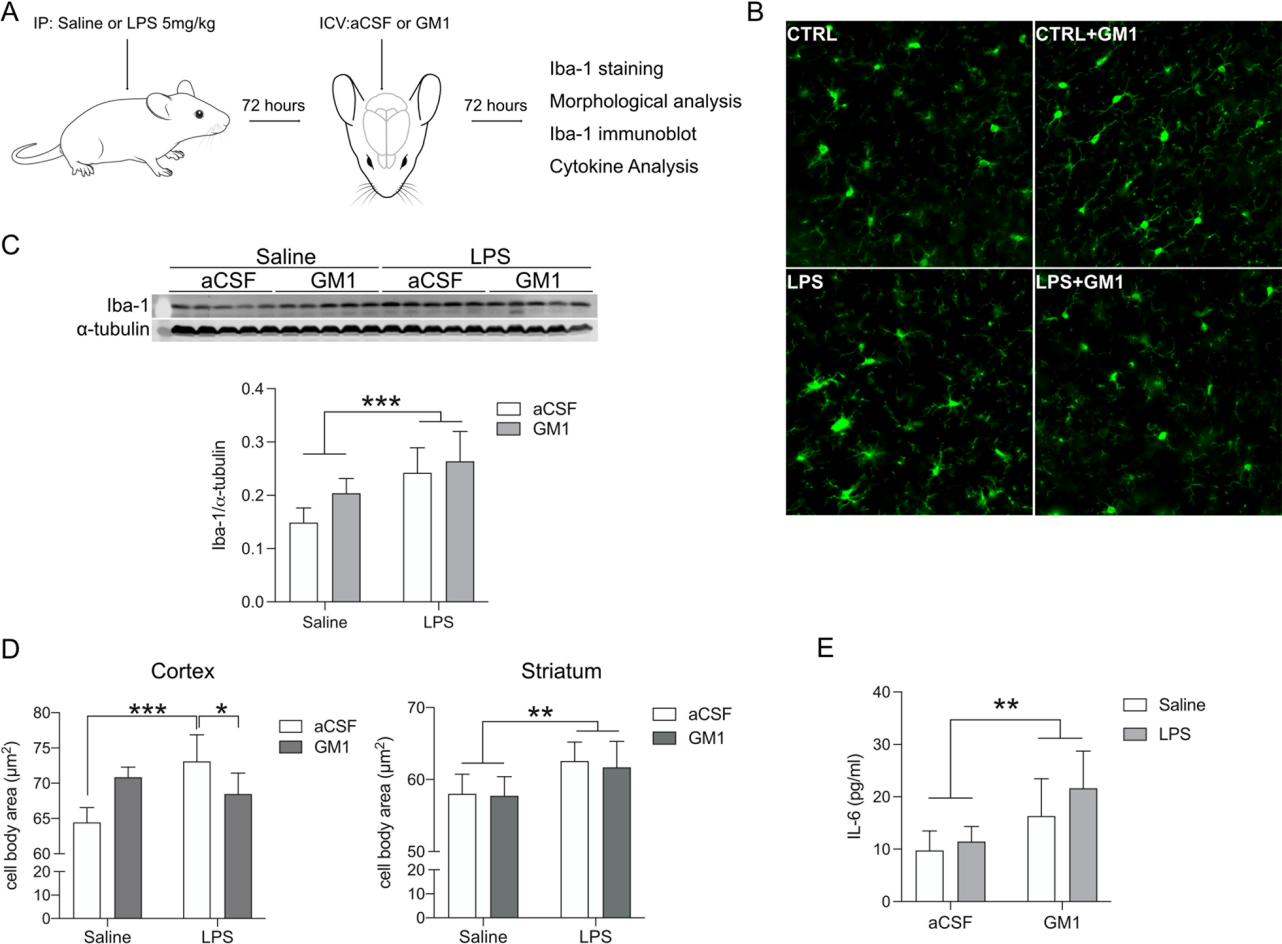
GM1 effects on brain microglia in vivo, after peripheral administration of LPS. **A** Schematic representation of in vivo administration of GM1 after LPS-induced systemic inflammation in mice (*N* = 5 per treatment). **B** Representative images of Iba-1 stained microglia in the cortex of control animals infused with vehicle (CTRL) or GM1 (CTRL + GM1), and in animals treated with LPS and subsequently infused with vehicle artificial cerebrospinal fluid (LPS) or GM1 (LPS + GM1). **C** Immunoblot in whole brain lysates and densitometric analysis show LPS-dependent increase in Iba 1 expression. **D** Cortical and striatal microglia (Iba-1^+^ cells) cell body area quantified with MetaXpress software. GM1 administration significantly decreases cell body area in the cortex, but not in the striatum. **E** IL-6 protein levels in whole mouse brain homogenate. aCSF, artificial cerebrospinal fluid. Bars show mean values ± STDEV. Two-way ANOVA with Tukey’s multiple comparisons test; *p < 0.05, **p < 0.01, ***p < 0.001

To investigate the effects of GM1 in vivo*,* we first injected C57BL6J mice intraperitoneally with LPS (5 mg/kg) or saline solution to induce neuroinflammation [52] and 3 days later we started to infuse GM1 or vehicle (artificial cerebrospinal fluid, aCSF) intraventricularly, for 3 more days (Fig. 2A). At the end of treatment, we analyzed the number of Iba-1 positive cells and their morphology in two main brain regions, the cortex and the striatum. Representative microscopy images of Iba-1-immunoreactive microglia are shown in Fig. 2B. In animals treated with LPS, we did not detect statistically significant changes in microglia cell numbers (Additional file 1: Fig. S3A). The number of microglia cell processes was also comparable in LPS-treated and control cortex, but slightly increased in the striatum (Additional file 1: Fig. S3B). The number of branches was similar across all experimental groups (Additional file 1: Fig. S3C) and so were levels of TNF (Additional file 1: Fig. S3D). However, we detected an increase of Iba-1 protein expression in whole brain homogenate of animals treated with LPS (Fig. 2C), as well as an increase in microglial cell body area—a sensitive measure of microglia activation [53–56]—which was brought back to control levels by GM1 (Fig. 2D). GM1 treatment also increased the levels of IL-6 in both control and LPS-treated animals (Fig. 2E).

**Fig. 3:**
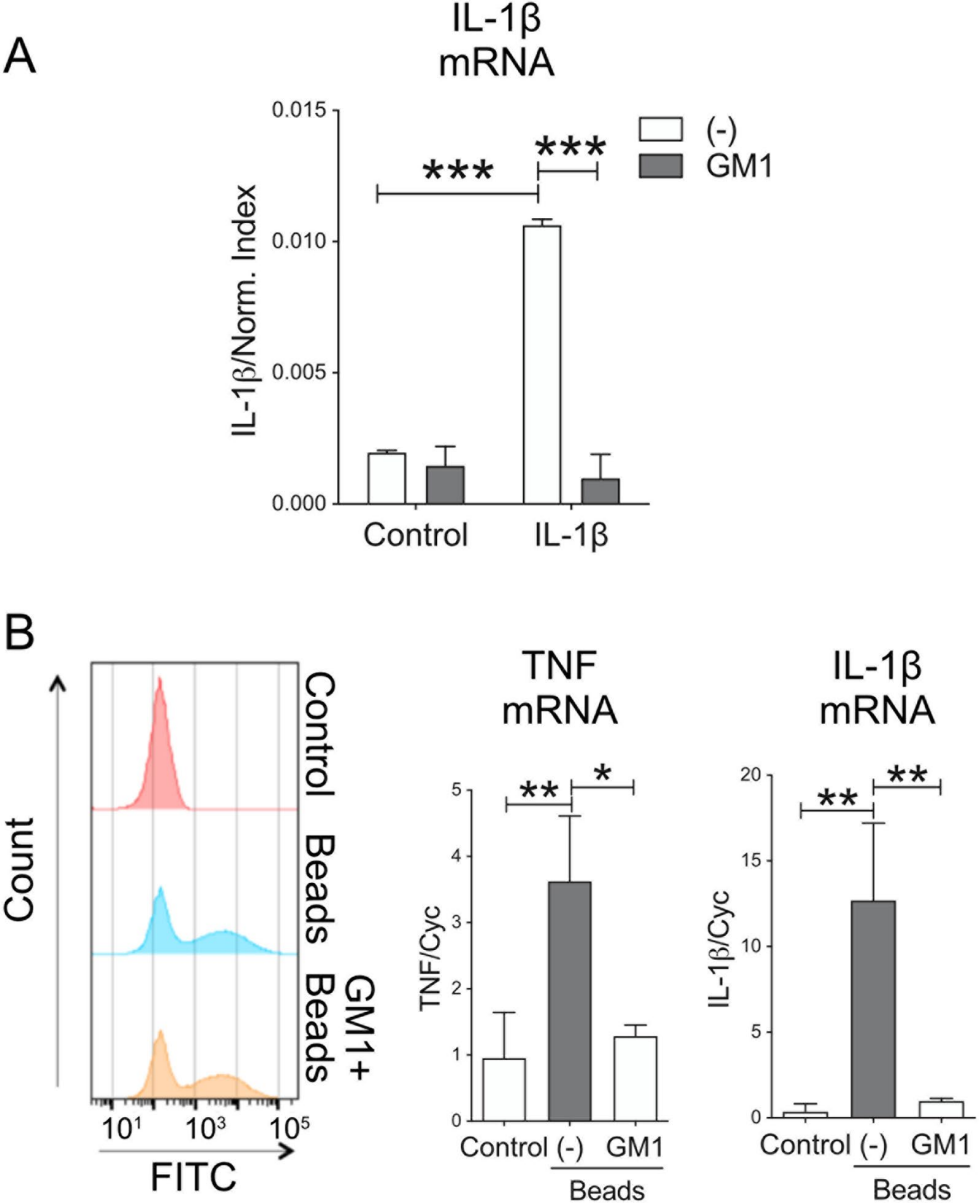
Pre-incubation of microglia with GM1 decreases pro-inflammatory activation triggered by IL-1β and by phagocytosis of polystyrene beads. **A** Mouse primary microglia were pre-incubated with GM1 (50 µM) for 1 h and then stimulated with IL-1β (5 ng/ml) for 24 h. GM1 blocked upregulation of IL-1β mRNA expression. **B** Phagocytosis of latex beads by BV-2 cells pre-treated with GM1 for 2 h. Representative histograms of beads uptake are shown on the left. GM1 pre-treatment did not affect uptake. Graphs show mRNA expression for IL-1β and TNF upon bead phagocytosis. Data are mean values ± STDEV of 3 independent experiments. Two-way ANOVA with Tukey’s multiple comparisons test was used in A. One-way ANOVA with Sidak’s multiple comparisons test was used in B. *p < 0.05, **p < 0.01, ***p < 0.001

To determine whether GM1 administration dampens inflammatory responses triggered by stimuli other than LPS, we exposed primary cultures of mouse microglia to IL-1β, to mimic a physiological response to a milder stimulus relevant to neurodegenerative and neuroinflammatory conditions [57, 58]. Administration of GM1 inhibited the transcriptional response to IL-1β stimulation, as measured by the expression of IL-1β (Fig. 3A).

GM1 also decreased the expression of pro-inflammatory genes following BV2 cell exposure to latex beads—another stimulus known to activate inflammatory responses [59]—without affecting the amounts of beads that were phagocytosed by the cells (Fig. 3B). Altogether, these data suggest that administration of GM1 dampens microglial inflammatory responses to different stimuli, even *after* exposure to the inflammatory triggers.

### The anti-inflammatory properties of GM1 depend on the sialic acid residue and the presentation of the glycan headgroup.

**Fig. 4:**
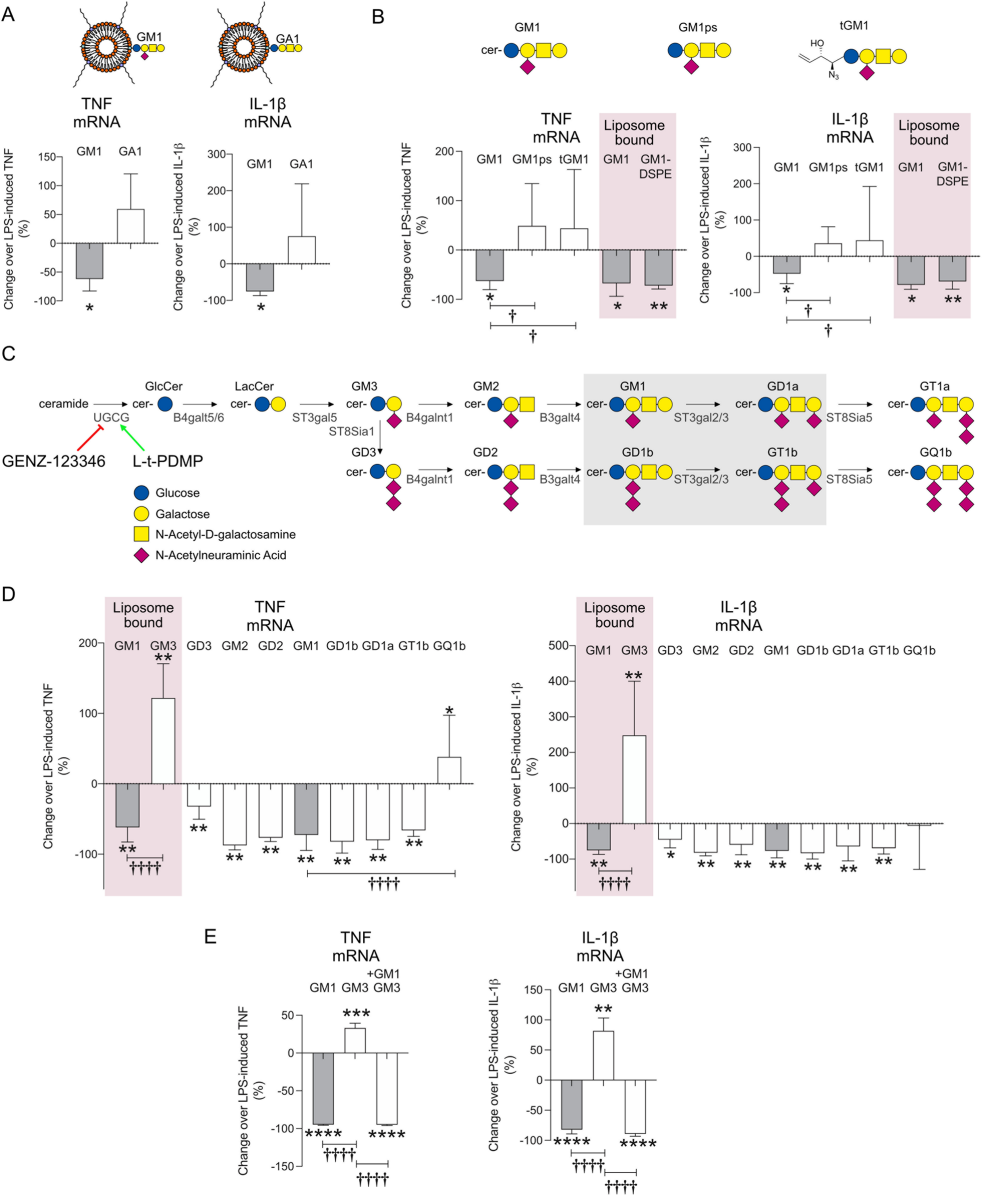
Anti-inflammatory effects of GM1 require the presence of sialic acid and a lipid tail and are shared by other gangliosides, but not GM3 or GQ1b. **A** Mouse primary microglia were stimulated for 3 h with LPS (100 ng/ml), washed and further incubated with GM1- or GA1-loaded liposomes (200 µM liposome concentration). Naked liposomes were used as a control. TNF and IL-1β mRNA levels were measured by qPCR. Data are presented as percentage expression change compared to activated microglia incubated with control naked liposomes (*N* = 3–4). **B** Mouse primary microglia were stimulated with LPS and washed as in A, prior to incubation with GM1, GM1-pentasaccharide (GM1ps) or truncated azido-GM1ps (tGM1) (50 µM each) for 8 h. In parallel experiments, activated microglia were incubated with control naked liposomes or GM1- or GM1-DSPE-loaded liposomes (200 µM each—pink shaded area) for 8 h (*N* = 3–4). **C** Simplified scheme of the ganglioside biosynthetic pathway and related enzymes. L–*t*-PDMP is an activator and GENZ-123346 is an inhibitor of UDP-glucose ceramide glucosyltransferase (UGCG). The shaded grey area highlights the major brain gangliosides. Glucose: blue circle; galactose: yellow circle; N-acetylgalactosamine: yellow square; N-acetylneuraminic acid: purple diamond. **D** Mouse primary microglia stimulated with LPS (100 ng/ml) for 3 h were washed and further incubated with naked liposomes, GM1- or GM3-loaded liposomes (200 µM each—pink shaded area) or the indicated gangliosides (all at 50 µM in PBS) for 8 h. Data are presented as the percentage expression change compared to activated microglia incubated with vehicle controls (naked liposomes or PBS) (*N* = 3–7). **E** LPS-stimulated microglia were washed and incubated with GM1- or GM3-containing liposomes (200 µM), or with GM3-liposomes in the presence of GM1 in micellar form (50 µM) for 8 h. Co-administration of GM1 abrogated the pro-inflammatory effects of GM3. Data are presented as percentage gene expression change over LPS-activated microglia treated with vehicle. Mean values ± STDEV are shown. A two-tailed *t*-test was used to compare the effect of each treatment to their respective vehicle controls. *p < 0.05, **p < 0.01. One-way ANOVA with Sidak’s multiple comparisons test was used in **B, D and E** to compare the effect of GM1 to other gangliosides. ^✝^*p* < 0.05, ^✝✝✝✝^*p* < 0.0001

To shed light on the structural determinants of the anti-inflammatory effects of GM1, we first tested the requirements for the sialic acid in the GM1 glycan headgroup. Asialo-GM1 (GA1) is poorly soluble in aqueous solutions. Therefore, we delivered both GA1 and GM1 with liposome carriers (200 µM liposomes as measured by total lipid, corresponding to a ganglioside concentration of 6 µM in the culture medium). Liposome-embedded GM1, but not GA1, was as effective as free GM1 at reducing the expression of TNF and IL-1β induced by LPS (Fig. 4A). The trend towards increased expression of TNF and IL-1β in LPS-stimulated cells treated with GA1 was not statistically significant, and GA1 alone (in liposomes) did not elicit any inflammatory response per se, in the absence of LPS (data not shown). Therefore, the sialic acid residue in the ganglioside headgroup is necessary to decrease the TLR-4 signaling induced by GM1.

The pentasaccharide of GM1 (GM1ps, 50 µM)—which includes the sialic acid residue—was not sufficient to reproduce the anti-inflammatory effects of the full GM1 molecule (Fig. 4B). This suggests that the ceramide tail of the ganglioside is required for the anti-inflammatory response. A potential explanation for this finding is that the amide, the hydroxyl and/or the alkene groups of the ceramide contribute to the ganglioside interaction with membrane receptors/partners [60, 61] that activate the anti-inflammatory signaling cascade. Alternatively, a clustered presentation of the ganglioside in micelles (as in our studies) or liposomes, or its ability to be incorporated into membranes—both of which depend on the presence of a lipophilic tail—might be required for signaling and/or ganglioside internalization by microglia cells. To discriminate between these two possibilities, we treated LPS-activated microglia with (1) a soluble GM1 analogue (truncated GM1, or tGM1) that lacked the hydrocarbon chains, but included the hydroxyl and the alkene groups of the sphingoid base (Additional file 1: Fig. S4, structure I); or (2) with liposomes carrying the same analogue linked to the phospholipid 1,2-stearoyl-phosphatidylethanolamine (DSPE) (Additional file 1: Fig. S4, structure III). Like GM1ps, the tGM1 analogue did not have anti-inflammatory activity (Fig. 4B). On the other hand, the DSPE-conjugated analogue embedded within liposomes did produce an anti-inflammatory effect (Fig. 4B, pink-shaded area), suggesting that a clustered presentation of the ganglioside and/or its incorporation into membranes is required for the anti-inflammatory effects of GM1.

### Several major gangliosides have anti-inflammatory properties, but GM3 and GQ1b are pro-inflammatory

To assess the specificity of the glycan headgroup and to determine whether other major gangliosides (Fig. 4C) share the anti-inflammatory properties of GM1, we compared the effects of different gangliosides on mouse primary microglia stimulated with LPS. In all cases, gangliosides were administered after microglia stimulation with LPS for 3 h, as indicated above for GM1. GM3 was prepared in liposomes, due to its low solubility in an aqueous solution, while other gangliosides were resuspended in PBS. Contrary to liposome-bound GM1, liposome-bound GM3 increased the expression of IL-1β and TNF mRNA in cells activated with LPS (Fig. 4D). GQ1b had similar effects on TNF expression (Fig. 4D), while it did not alter IL-1β expression. Neither GM3, nor GQ1b had pro-inflammatory effects on naïve cells (i.e., in the absence of LPS—Additional file 1: Fig. S5), suggesting that these two gangliosides enhance LPS-induced TLR signaling, rather than activating microglia by themselves. All other ganglioside tested, GD3, GD2, GM2, GD1a, GD1b, and GT1b, decreased LPS-induced TNF and IL-1β expression to an extent similar to GM1 (Fig. 4D). To determine whether GM1 could antagonize GM3, LPS-stimulated microglia were co-treated with GM3-embedded liposomes and GM1. The presence of GM1 blocked the pro-inflammatory effects of both LPS and GM3 (Fig. 4E).

### Endogenous ganglioside levels modulate the response of microglia to pro-inflammatory stimulation

**Fig. 5:**
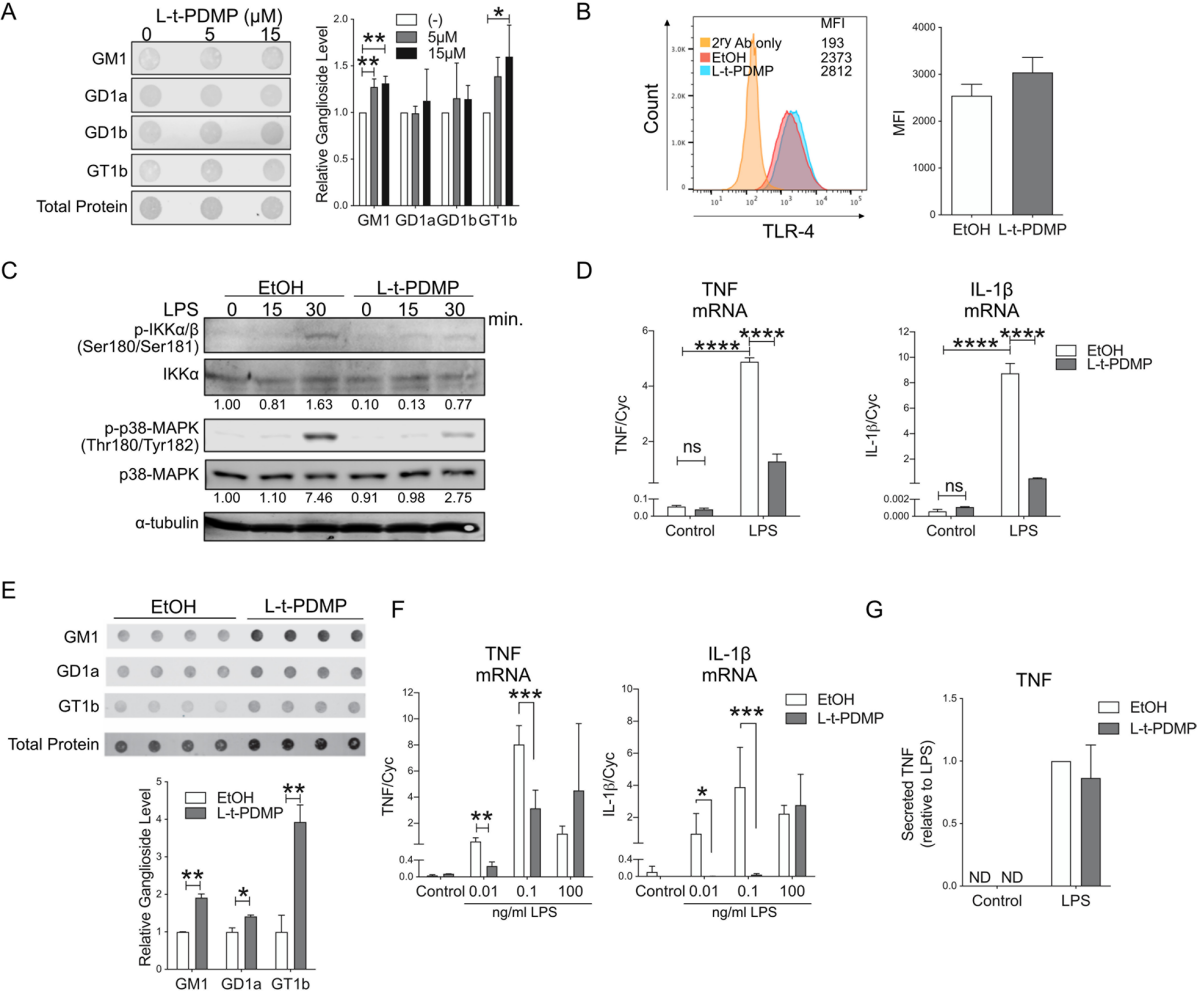
Stimulation of the ganglioside biosynthetic pathway with L–t-PDMP decreases pro-inflammatory microglia activation.** A** BV2 cells were incubated for 72 h with the indicated concentrations of L–*t*-PDMP to increase ganglioside synthesis. A representative dot-blot and quantification of cellular ganglioside levels before and after treatment are shown (N = 3). **B** Representative histogram and relative flow cytometry quantification (mean fluorescence intensity, MFI) of TLR-4 present at the plasma membrane of BV-2 cells after treatment with 10 µM L–*t*-PDMP for 72 h. **C** Representative immunoblot showing phospho-IKK and phospho-p38 MAPK levels in BV2 cells stimulated with LPS (100 ng/ml) for the indicated time, after cell treatment with L–*t*-PDMP (15 µM, 72 h). The numbers under the blots show fold-change over untreated control, after normalization for total IKK or p38-MAPK levels. The experiment was repeated twice with similar results. **D** Expression of TNF and IL-1β mRNA in BV-2 cells treated as indicated above (*N* = 3). **E** Dot-blot analysis of ganglioside levels in murine primary microglia incubated with L–*t*-PDMP (10 µM for 72 h). **F** Expression of IL-1β and TNF mRNA in primary microglia treated for 72 h with 10 µM L–*t*-PDMP and stimulated with the indicated concentrations of LPS for 6 h (*N* = 3). **G** TNF secretion by murine microglia after treatment with L–*t*-PDMP and stimulation with the indicated concentrations of LPS (*N* = 3). Data shown are mean values ± STDEV. One-way ANOVA with Sidak’s multiple comparisons test was used in **A**; two-tailed *t* test was used in **B** and **E**; two-way ANOVA with Tukey’s multiple comparisons test was used in **D**, **F** and **G**. *p < 0.05, **p < 0.01, ***p < 0.001, ****p < 0.0001

To determine whether *endogenous* ganglioside levels can affect the response of microglia to pro-inflammatory stimuli, we used the compound L–*t*-PDMP to enhance the activity of microglial UDP-glucose ceramide glucosyltransferase (UGCG) [62] and increase cellular ganglioside levels (Fig. 4C). In BV2 cells, treatment with L–*t*-PDMP (5–15 µM) resulted in a significant increase in the levels of gangliosides GM1 and GT1b (Fig. 5A), two of the four most abundant gangliosides in the brain. Although the reduction in the abundance of these gangliosides was associated with a decrease in total cellular levels of TLR-4 protein (Additional file 1: Fig. S6A), it did not affect cell surface expression of the receptor (Fig. 5B). In L–*t*-PDMP-treated cells, activation upon exposure to LPS (100 ng/ml) was attenuated, as shown by decreased phosphorylation of IKK and p38-MAPK (Fig. 5C). Concomitantly, we observed a significant reduction in LPS-induced expression of IL-1β and TNF mRNA (Fig. 5D). Similar results were obtained in mouse primary microglia, where stimulation of ganglioside synthesis with L–*t*-PDMP resulted in higher cellular levels of GM1, GD1a and GT1b (Fig. 5E), concomitantly with an attenuated response (decreased levels of IL-1β and TNF mRNA) at low LPS concentrations (0.01 and 0.1 ng/ml) (Fig. 5F). At these LPS concentrations, we were not able to detect TNF released in the medium. At higher LPS concentrations (100 ng/ml), L–*t*-PDMP-treated and untreated cells responded similarly (Fig. 5F, G). Overall, our data suggest that increasing endogenous levels of microglial gangliosides attenuates microglia response to an inflammatory stimulus, at least to a certain extent.

**Fig. 6:**
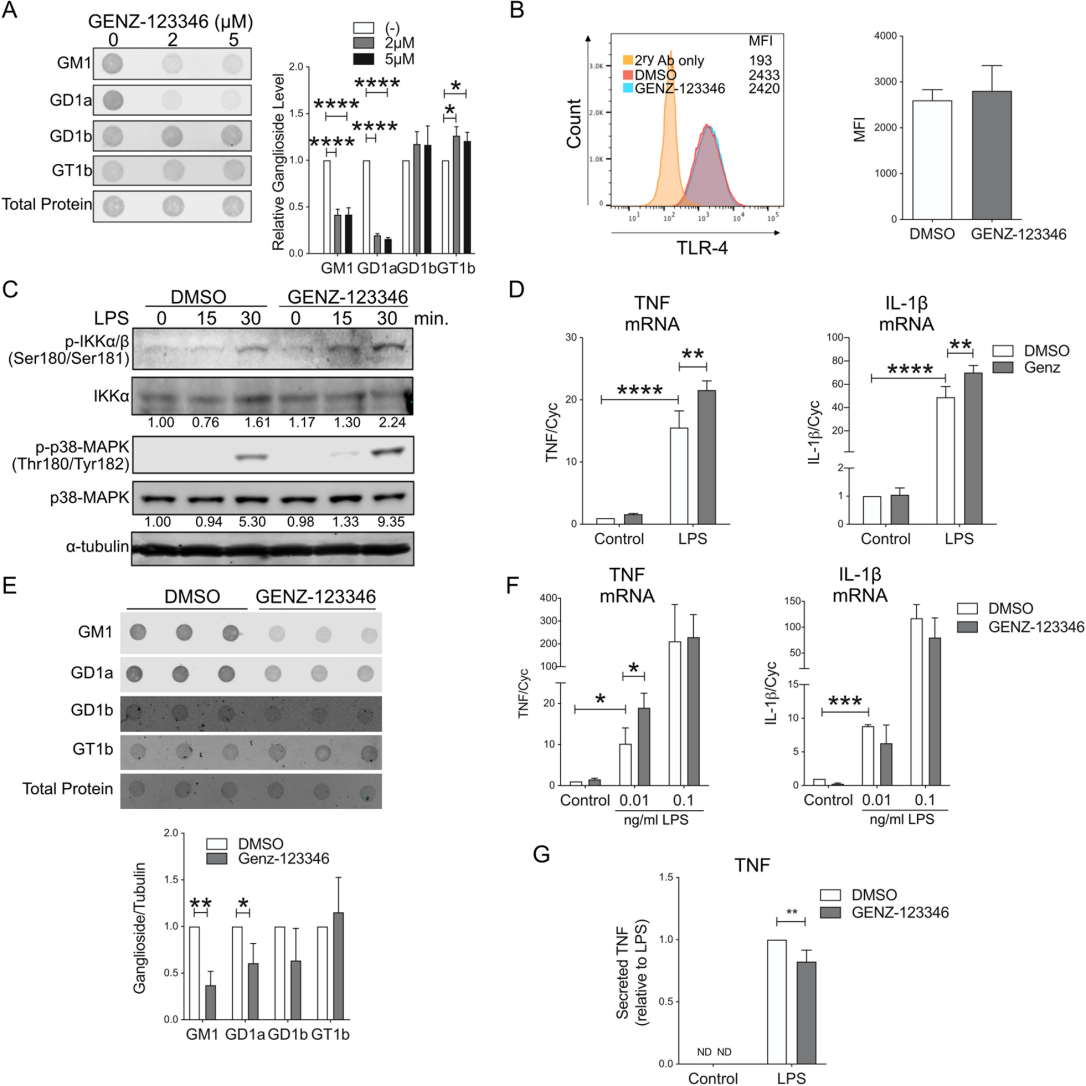
Inhibition of the ganglioside biosynthetic pathway enhances microglial response to LPS. **A** Dot-blot and relative quantification of cellular ganglioside levels in BV2 cells treated with the indicated concentrations of GENZ-123346 for 72 h shows a significant decrease in levels of GM1 and GD1a (*N* = 3). **B** Representative histogram and relative flow cytometry quantification (mean fluorescence intensity, MFI) of TLR-4 present at the plasma membrane of BV-2 cells after treatment with 10 µM GENZ-123346 for 72 h. **C** Representative immunoblot showing increased phosphorylation of IKK and p38 MAPK in BV-2 cells treated with GENZ-123346 and stimulated with LPS (100 ng/ml) for the indicated time. The numbers under the blots indicate the fold-change of p-IKK and p-p38-MAPK compared to untreated control, after normalization over total IKK or p38-MAPK protein levels. **D** Expression of IL-1β and TNF mRNA in BV-2 cell stimulated with LPS (100 ng/ml) after 72 h incubation with GENZ-123346 (5 μM) (*N* = 3). **E** Dot-blot and quantification of ganglioside levels in primary mouse microglia incubated with 10 µM GENZ-123346 for 72 h show a significant decrease in the levels of both GM1 and GD1a (*N* = 3). **F** Expression of IL-1β and TNF mRNA in mouse microglia treated with GENZ-123346 as in **E**, and after stimulation with the indicated concentrations of LPS for 6 h (*N* = 3). **G** TNF secretion by control and GENZ-123346-treated cells upon stimulation with LPS (1 ng/ml) (*N* = 3). Data shown are mean values ± STDEV. One-way ANOVA with Sidak’s multiple comparisons test was used in **A**; two-tailed *t* test was used in **B** and **E**; two-way ANOVA with Tukey’s multiple comparisons test was used in **D**, **F** and **G**. *p < 0.05, **p < 0.01, ***p < 0.001, ****p < 0.0001

In neurodegenerative conditions, such as PD and HD, brain levels of gangliosides are decreased [27, 29, 63–68]. Furthermore, in our experiments, treatment of mouse microglia with LPS resulted in decreased GM1 and GT1b levels, although levels of GD1a were increased (Additional file 1: Fig. S7). Therefore, we next explored the potential impact that a decrease in the microglial levels of gangliosides would have on microglia activation. We used the compound GENZ-123346 [69] to specifically inhibit the activity of UGCG in BV2 cells and primary microglia and reduce cellular ganglioside levels (Fig. 4C). In BV2 cells, treatment with GENZ-123346 resulted in > 50% decrease in GM1 and GD1a levels, although, unexpectedly, the amount of the complex ganglioside GT1b increased slightly (Fig. 6A). These changes were accompanied by increased phosphorylation of IKK and p38-MAPK upon cell stimulation with LPS compared to cells with normal expression of gangliosides (Fig. 6C), and a modest but significant increase in the expression of pro-inflammatory cytokines (Fig. 6D). Total cellular and cell surface expression of TLR-4 were not significantly affected by the treatment (Fig. 6B and Additional file 1: Fig. S6B). Like BV2 cells, treatment of primary microglia with GENZ-123346 resulted in decreased GM1 and GD1a levels (Fig. 6E) and increased transcription of TNF after microglia stimulation with LPS (0.01 ng/ml for 6 h) (Fig. 6F). No effects on IL-1β transcription were observed in these experiments (Fig. 6F). Surprisingly, the TNF mRNA increase was not accompanied by a corresponding increase in the amount of TNF secreted in the medium, which, instead, was slightly decreased in cells treated with GENZ-123346 (Fig. 6G).

## Discussion

In this study, we analyzed the effects of pharmacological treatments that modulate cellular ganglioside levels on microglia activation by inflammatory stimuli. Of all gangliosides, GM1 is the most widely investigated for its neuroprotective properties in models of neurological conditions, including stroke [70, 71], PD [72, 73], and HD [27, 38, 39]. While there is evidence that GM1 can activate protective mechanisms in neurons [74–76], it was not known whether GM1 might also affect microglia functions. This is an important gap in knowledge, since aberrant pro-inflammatory microglia activation contributes to disease pathogenesis and/or progression in all diseases listed above [8, 11, 14, 77–79].

Our work provides evidence, for the first time, that GM1 exerts a strong anti-inflammatory effect in different microglia culture systems exposed to various pro-inflammatory stimuli, including LPS, IL-1β and phagocytosis of latex beads, while no effects of the ganglioside were observed in unstimulated conditions. Importantly, we have shown that these anti-inflammatory effects are not limited to murine models, but also extend to human microglia and are, therefore, directly relevant to human pathophysiology.

Our data are in contrast with previous reports of activation of the p38 MAPK pathway and morphological changes suggestive of microglial activation upon microglia exposure to GM1 [80]; or that showed secretion of pro-inflammatory TNF and NO by microglia treated with a mix of brain gangliosides containing GM1 [81–83]. Whether modality of administration and/or source of gangliosides might be responsible for these discrepancies is not known. On the other hand, our findings are in line with and expand on studies performed on myeloid cells, which showed that GM1 and other gangliosides attenuate the response of human monocytes, THP-1 cells and RAW 264.7 macrophages to LPS [84–86] and amyloid-β-peptide [87, 88].

In an in vivo model that mimics the interplay existing between systemic inflammation and neuroinflammation and neurodegeneration [52, 89], intracerebroventricular infusion of GM1 after peritoneal injection of LPS resulted in decreased microglia cell body area, which is a sensitive marker of microglia activation [53–56]. At the timepoint analyzed in our experiments, no other major signs of inflammation were observed in the mouse brains, and the number of microglia cells was similar in animals that received LPS or saline. Nevertheless, the expression of the microglial marker Iba-1 (ionized calcium-binding adapter molecule 1) was increased in tissue from animals treated with LPS. Iba1 is a calcium-binding protein important for membrane ruffling, phagocytosis, and microglia motility [90, 91]. Expression changes that are not accompanied by an increase in microglia cell number might reflect microglia states and functions that are independent from inflammatory activation and that were not affected by GM1 in our model system.

Contrary to previous studies [89], in our experiments, brain TNF levels were not significantly altered by systemic LPS treatment, or had returned to basal expression at the timepoint when we performed cytokine analysis (6 days after LPS injection). Consequently, we could not assess the ability of GM1 to decrease TNF expression in vivo. On the other hand, we found that treatment with GM1 was responsible for an increase in brain levels of IL-6, independently from LPS stimulation. This is notable as, besides its role in inflammation [92, 93], IL-6 plays several other functions [94]: it has neurotrophic activity [95, 96], is involved in the regeneration of peripheral nerves [97] and the differentiation of oligodendrocytes [98], and exerts neuroprotective and reparative activities in models of neuronal injury [89, 99]. Whether increased expression of IL-6 contributes, at least in part, to the neuroprotective and restorative effects of GM1 in models of neurodegeneration remains to be determined. The source of this IL-6 increase is also currently unknown, since various brain cells can secrete this cytokine, including microglia, endothelial cells and neurons, [94].

The mechanism underlying the anti-inflammatory effects of GM1 is currently under investigation in our laboratory. Previous studies have shown that GM1 might bind to some LPS serotypes and potentially decrease binding to TLR-4 when pre-incubated with the bacterial toxin [100, 101]. The LPS serotype used in this study (*E. coli* O55:B5), however, does not bind GM1 [101]. In other studies, incubation of microglia with GM1 [102] or with a mix of brain gangliosides [102] resulted in downregulation of TLR-4 expression. Although in our experiments we observed slightly decreased levels of total cellular TLR-4 in microglial BV2 cells incubated for 24 h with GM1, the levels of plasma membrane receptor available for binding were not significantly changed by the ganglioside. In addition, we found that GM1 could still curtail microglia inflammatory responses after microglia had been activated and after LPS removal. While this does not exclude potential modulatory effects of GM1 on TLR-4 signaling, it suggests that the ganglioside activates a “shut-off” pathway that helps to restore homeostatic conditions upon exposure to an inflammatory stimulus. This hypothesis is further supported by our observation that GM1 decreases pro-inflammatory microglia responses triggered by IL-1β or by phagocytosis of latex beads, which are known to activate the NLRP3 inflammasome and downstream inflammatory response [59].

Our experiments revealed that the presence of the sialic acid residue in the glycan headgroup of GM1 is essential for attenuating the response of microglia to LPS. This finding points to the potential involvement of a sialic acid-binding receptor as a mediator of the anti-inflammatory effects of GM1 and other gangliosides. Potential candidates could be sialic acid-binding immunoglobulin-like lectins (Siglecs). Many of these proteins contain immunoreceptor tyrosine-based inhibitory motifs (ITIMs) that recruit tyrosine phosphatases, such as SHP1 and SHP2 to decrease pro-inflammatory stimulation of immune cells [103, 104]. Some Siglecs were also shown to interact with and modulate the activity of TLRs [105]. Future investigations will address the hypothesis of Siglecs involvement by GM1 and other gangliosides.

In our studies, neither the soluble oligosaccharide portion of GM1 nor a soluble truncated GM1 analogue that included the alkene and hydroxyl groups of the sphingoid base (but had no hydrocarbon chains) were able to reproduce the anti-inflammatory activity of GM1. However, if the truncated analogue was attached to DSPE to mediate insertion into liposomes, the anti-inflammatory properties were restored. These data suggest that the hydrophobic tail of the ganglioside is required for its anti-inflammatory activity in addition to the glycan headgroup. A potential explanation for this requirement is that the hydrophobic tail (ceramide or DSPE) allows the ganglioside to be incorporated into cell membranes from which it can engage in *cis*-interactions and signaling. An alternative explanation is that the “presentation” of the glycan headgroup in a clustered configuration, as provided by the ganglioside arrangement in micelles or liposomes, is necessary to efficiently engage microglial receptors (trans-interactions) and inhibitory signaling pathways. This second hypothesis is in line with evidence that receptor clustering and multivalent binding are often required for signaling, including in the case of Siglecs activation [104].

Our finding that the gangliosides hydrophobic tail is required for anti-inflammatory effects is in contrast with the ability of the soluble GM1 oligosaccharide to mimic other ganglioside biological functions, including binding and activation of the tropomyosin receptor kinase TrkA [106]. It also highlights the heterogeneity of mechanisms used by glycolipids to interact with and modulate the activity of protein partners and signaling pathways [21].

The anti-inflammatory properties of GM1 were shared by all other major gangliosides, except for GM3 and GQ1b, which had opposite effects. The underlying reasons are not known. Of note, GM3 is the simplest and smallest (in terms of headgroup size), while GQ1b is the most complex and the largest among the gangliosides tested in this study. As the size, charge and structure of the glycan headgroup significantly affect ganglioside propensity for segregation and membrane curvature, as well as rigidity and spatial conformations of the sugar residues in the ganglioside headgroups [107], it is plausible that these gangliosides at the two extremes of ganglioside complexity might not present the optimal combination of chemical, steric and biophysical properties required to exert anti-inflammatory effects in our model systems.

Together with the requirement for sialic acid residues in the glycan headgroup, our findings support the hypothesis that a specific, glycan-dependent ganglioside-protein interaction—and not just potential changes in membrane fluidity and lipid rafts due to the increased ganglioside concentration at the membrane—is necessary to activate anti-inflammatory pathways in our models.

While in BV2 cells, treatment with L–*t*-PDMP (to increase endogenous ganglioside levels) recapitulated the effects of exogenous GM1 administration, in primary microglia L–*t*-PDMP decreased expression of pro-inflammatory genes only at lower (but still physiologically relevant) LPS concentrations. This is likely due to higher levels of cellular GM1 achieved upon administration of exogenous GM1 compared to the pharmacological stimulation of UCGC activity.

Decreasing endogenous ganglioside levels with GENZ-123346 resulted in opposite effects, including a stronger activation of the NFkB and p38 MAPK pathways and increased transcription of TNF mRNA upon cell stimulation with LPS. Secretion of TNF in the culture medium, however, was not affected by GENZ-123346 treatment. This might be due to the specific time-frame of our experiments, which might have allowed for the detection of early transcriptional changes but not subsequent and later effects at a protein level; or to the presence of post-transcriptional regulatory mechanisms [108–110] that might not be directly affected by gangliosides. In any case, the cumulative evidence obtained in our study using two different cell models suggest that inhibition of ganglioside synthesis and decreased levels of gangliosides in microglia make the latter more responsive to pro-inflammatory stimulation. We speculate that these changes might contribute to the acquisition of a maladaptive inflammatory phenotype in the context of those neurodegenerative conditions where gangliosides are affected [27–30]. Interestingly, in mouse microglia exposed to LPS for 24 h we found changes in the levels of 3 major gangliosides we measured, with mildly decreased levels of GM1 and GT1b, and increased levels of GD1a. This suggests that the ganglioside profile of microglia might be subjected to modulation (whether by changes in ganglioside synthesis or by ganglioside remodelling) depending on microglia state. Future studies that analyze the full spectrum of gangliosides in different microglia states will be required to assess whether state-dependent changes in the microglial ganglioside profile affect other microglia functions.

## Conclusions

Our data suggest that microglial gangliosides play an important role in the regulation of the response of microglia to inflammatory stimuli, and highlight the specific glycan structures required. Our studies provide insights into the beneficial roles of GM1 in neurodegenerative diseases and demonstrate that the ganglioside can target inflammatory microglia in addition to neurons.

Future studies will address the underlying mechanism(s) and will help to identify novel strategies to lower microglia activation in the context of neuroinflammatory conditions. Altogether, our data suggest that administration of exogenous GM1 elicits a potent cell-autonomous anti-inflammatory response in microglia, which might contribute to the neuroprotective activity of this ganglioside in models of neurodegeneration and neuroinflammation.

## Supplementary Information

**Additional file 1.** Additional methods for the analysis of cell viability and TLR-4 surface expression; additional figures S1-S7, including GM1 dose-response, cell survival after microglia incubation with exogenous GM1, TLR-4 protein expression, analysis of the effects of GM1 on Iba-1+ cell number and Iba-1 expression in vivo, synthesis and structure of GM1-DSPE, effects of GQ1b and GM3 on naive microglia, and cell ganglioside analysis after stimulation of microglia with LPS.

## Data Availability

All data generated or analysed during this study are included in this published article and its additional information files.

## Abbreviations

AD: Alzheimer’s disease
CNS: Central nervous system
GENZ-123346: N-[(1R,2R)-1-(2,3-Dihydrobenzo[b][1,4]dioxin-6-yl)-1-hydroxy-3-(pyrrolidin-1-yl)propan 2-yl] nonanamide
ERK: Extracellular signal-regulated kinase
GA1: Asialo-GM1
GM1ps: GM1-pentasaccharide
HD: Huntington’s disease
Iba-1: Ionized calcium-binding adapter molecule 1or Allograft inflammatory factor 1
IκBα: NFκB inhibitor alpha
IKK: IκB kinase
IL-1β: Interleukin-1β
IL-6: Interleukin-6
JNK: C-Jun N-terminal kinase
LPS: Lipopolysaccharide
L–*t*-PDMP: L-*threo*-1-phenyl-2-decanoylamino-3-morpholino-1-propanol·HCl
MAPK: Mitogen-activated protein kinase
NFκB: Nuclear factor NF-kappa-B
NLRP3: NACHT, LRR and PYD domains-containing protein 3
NO: Nitric oxide
PD: Parkinson’s disease
tGM1: Truncated GM1 azide
TNF: Tumor necrosis factor
TrkB: Tropomyosin receptor kinase B
TLR-4: Toll-like receptor 4
UGCG: UDP-glucose ceramide glucosyltransferase
