# Supplementary material for: Anti-inflammatory role of GM1 and other gangliosides on microglia
Source: J Neuroinflammation. 2022 Jan 6;19:9. doi: 10.1186/s12974-021-02374-x (PMC8739653; doi:10.1186/s12974-021-02374-x)

ADDITIONAL INFORMATION

Anti-inflammatory role of GM1 and other gangliosides on microglia

Danny Galleguillos[1,5,], Qian Wang[1,5,], Noam Steinberg[1,5], Asifa Zaidi[1,5], Gaurav Shrivastava[2], Kamaldeep Dhami[3], Gour C. Daskhan[4], Edward N. Schmidt[4], Zoë Dworsky-Fried[1], Fabrizio Giuliani[2,5], Matthew Churchward[3,5], Christopher Power[2,5], Kathryn Todd[3,5], Anna Taylor[1,5], Matthew S. Macauley[4,6], & Simonetta Sipione[1,5,#]

Department of Pharmacology[1], Department of Medicine[2], Department of Psychiatry[3], Department of Chemistry[4], Neuroscience and Mental Health Institute[5], Department of Medical Microbiology and Immunology[6] University of Alberta, Edmonton, AB, Canada

Corresponding Author
Simonetta Sipione
Department of Pharmacology
University of Alberta
9-21 Medical Sciences Building
Edmonton, Alberta
T6G 2H7 Canada
Phone: 780-492-5885
Fax: 780-492-4325
Email: ssipione@ualberta.ca

Keywords

Gangliosides, GM1, GENZ-123346, L-t-PDMP, liposomes, microglia, BV2 cells, LPS, inflammation

ADDITIONAL METHODS

Analysis of cell viability and TLR-4 surface expression by flow cytometry

Cell viability of primary microglia cells was measured using Annexin V-PE (BD Pharmigen 556421) and LIVE/DEAD™ Fixable Near-IR Dead Cells Kit (Invitrogen L34974). Briefly, cells were detached from the plates using TryPLE Express Enzyme (Gibco), washed with cold PBS and stained for 15 min with Annexin V-PE and LIVE/DEAD Fixable Far Red. After washing with PBS, fluorescence emission was detected using an Attune NxT Flow Cytometer (Invitrogen). To quantify TLR4 expression at the plasma membrane, BV-2 cells were incubated with 1% BSA on ice for 30 minutes and then stained with mouse anti-TLR4 (Abcam, ab22048) without fixation nor permeabilization. After washing, Alexa Fluor-488 anti-mouse IgG (Invitrogen) was added for 1 h on ice. Unstained controls were prepared by incubation with secondary antibodies only. Cells were analyzed with BD FACS Canto II (Becton Dickinson). Flow cytometry data were analyzed using FlowJo software.

ADDITIONAL FIGURE LEGENDS

Additional Figure S1

Administration of GM1 curtails pro-inflammatory microglia activation A) Dose-response analysis of the anti-inflammatory effect of GM1 in primary mouse microglia. Microglia were stimulated for 3h with LPS (100 ng/ml), after which LPS was washed out and cells were further incubated with GM1 at the indicated concentrations for 8 h (N=3-7). Mean values ± STDEV are shown. **B)** BV2 microglial cells were pre-incubated with GM1 (50μM) or vehicle for 2 h prior to stimulation with LPS (100 ng/ml for 6h). Upregulation of TNF and IL-1β mRNA expression was blunted in BV-2 cell pre-incubated with GM1 (N=3). **C)** Mouse primary microglia were pre-incubated with GM1 for 2 h followed by stimulation with LPS (100 ng/ml, 24h) and the expression of IL-1β, TNF and IκBα mRNA was measured (N=4-5). One-way ANOVA with Kruskal-Wallis multiple comparisons test was used in A. Two-way ANOVA with Tukey's multiple comparisons test was used in B and C. *p<0.05, **p<0.01, ***p<0.001, ****p<0.0001.

Additional Figure S2

Microglia survival and TLR-4 expression after incubation with exogenous GM1. A) Cell viability assay using LIVE/DEAD™ Fixable Near-IR Dead Cells stain and Annexin-V PE in mouse microglia

pre-incubated with GM1 (50 μM for 1h) and then stimulated with LPS (100 ng/ml for 24h), in the presence or absence of GM1. **B)** Cell viability assay in mouse microglia stimulated with LPS (100 ng/ml) for 3h, washed and then incubated with GM1 (50μM) for 8 additional hours. In both cases, treatment with GM1 did not increase microglia cell death. **C)** Representative histograms and relative flow cytometry quantification (mean fluorescence intensity, MFI) of TLR-4 present at the plasma membrane of BV-2 cells after treatment with GM1 (50 μM) for 4h and 24h. Bars show mean values ± STDEV. Two-tailed *t*-test. **D)** Representatives immunoblot and quantification of total TLR-4 expression in BV-2 cells treated with GM1 as indicated in **C** (N=4). One-way ANOVA with Dunnett's multiple comparisons test. *$p<0.05$.

Additional Figure S3

Analysis of Iba-1$^+$ cells and Iba-1 expression in LPS and GM1 treated mice. The number of Iba-1$^+$ cells per field **(A)**, the number of processes in Iba-1$^+$ cells (**B)** and the number of branching points in Iba-1$^+$ cells (**C)** were counted in cortical (top panels) and striatal (bottom panels) tissue from animals treated with LPS ± GM1. **D)** TNF levels in brain tissue of mice treated with LPS and GM1. Bars show mean values ± STDEV. Two-way ANOVA with Tukey's multiple comparisons test. **$p<0.01$, *** $p<0.001$

Additional Figure S4

Synthesis and structure of GM1-DSPE. I. Truncated GM1-azide (tGM1); **II.** Intermediate GM1-amine; **III.** GM1-DSPE. Percent yield for the various reactions is indicated on the right in parenthesis; H_2S: Hydrogen sulfide; Py: Pyridine; Et_3N: Triethylamine; DMF: Dimethylformamide; CE: Coupling Efficiency.

Additional Figure S5

Lack of pro-inflammatory effects by GQ1b and GM3 on naïve microglia cells. Mouse primary microglia were incubated with **A)** GQ1b (50 μM in PBS; N=7) for 8 h, or **B)** naked liposomes or GM3-loaded liposomes (200μM each; N=3). Graphs show mRNA expression for IL-1β and TNF after incubation with the indicated gangliosides.

Additional Figure S6

TLR-4 protein expression in BV2 cells upon activation or inhibition of the ganglioside biosynthetic pathway. A) Representative immunoblot and densitometric analysis of TLR-4

expression showing decreased levels of the receptor in BV2 cells treated with L-*t*-PDMP (N=6). **B)** Representative immunoblot and quantification of TLR-4 expression in BV-2 cells after 72 h incubation with GENZ-123346 (5µM) (N=5). Bars show mean values ± STDEV. A two-tailed *t*-test was used to compare the effect of L-*t*-PDMP or 10µM GENZ-123346 versus their respective vehicles.

Additional Figure S7

Expression of gangliosides GM1, GD1a and GT1b after stimulation of primary microglia with LPS. A) Representative dot blot and B) densitometric analysis of gangliosides GM1, GD1a and GT1b in primary mouse microglia treated for 24h with LPS (100 ng/ml) (N=5). Other gangliosides were not measured. Bars show mean values ± STDEV. A two-tailed *t*-test was used to compare the effect of LPS on the expression of the different gangliosides

ADDITIONAL FIGURES

Additional Figure S1

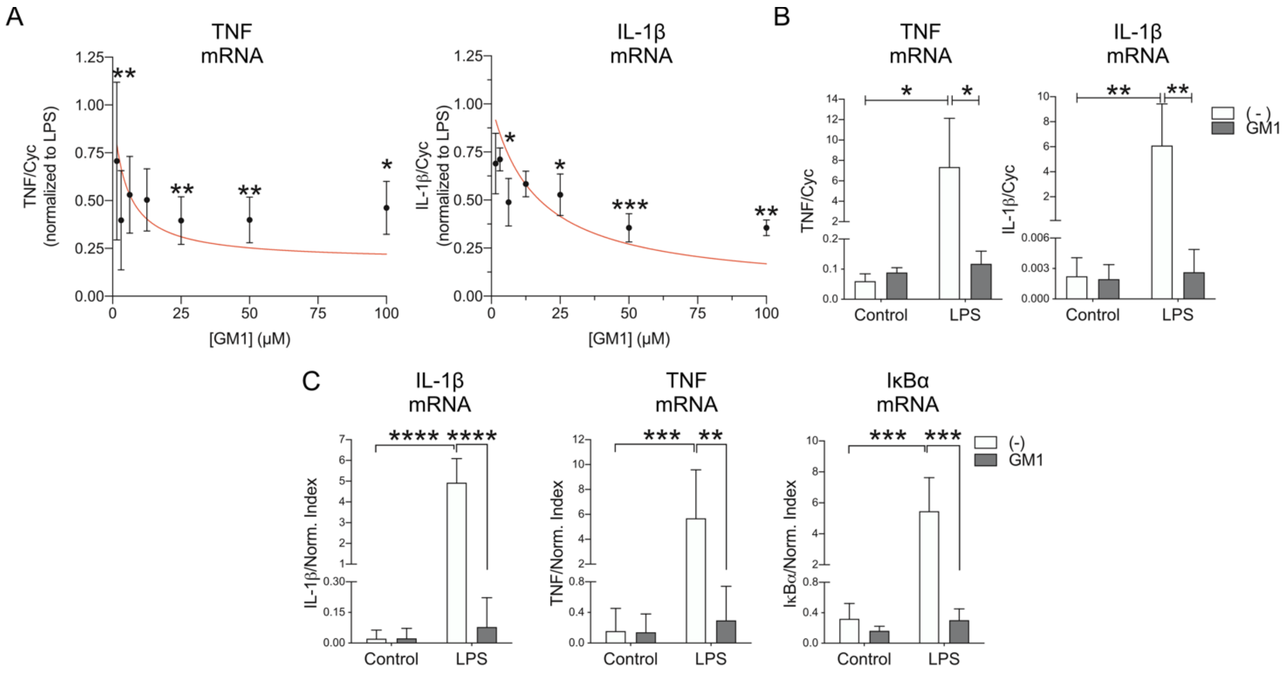

Additional Figure S2

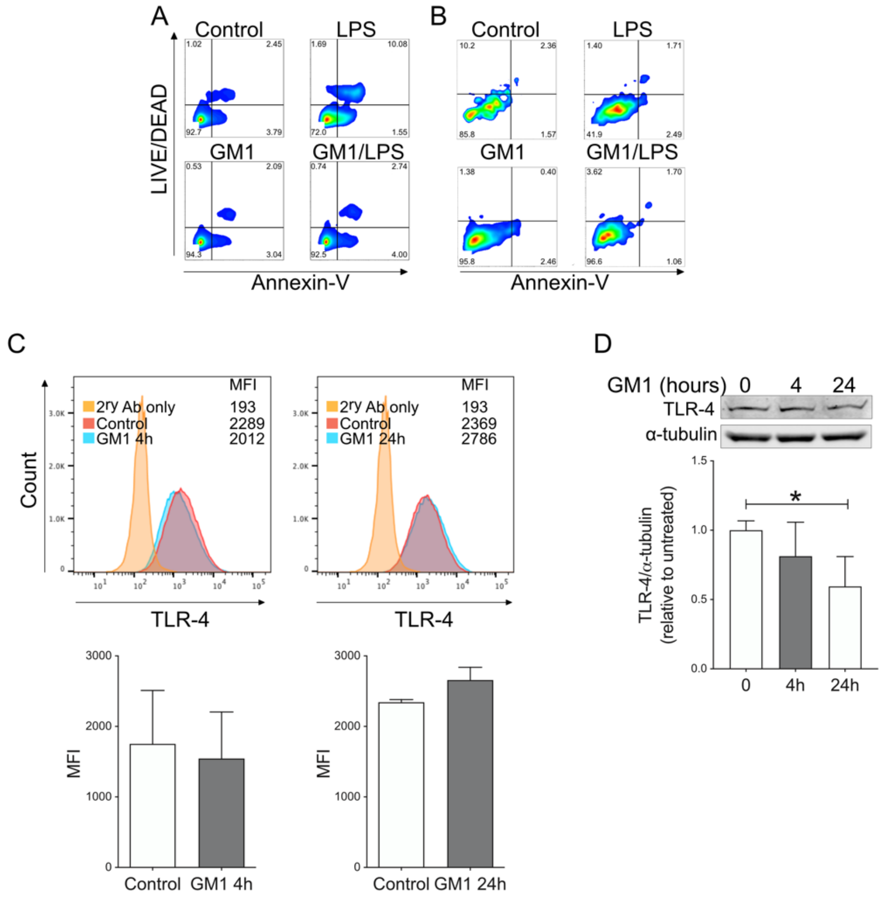

Additional Figure S3

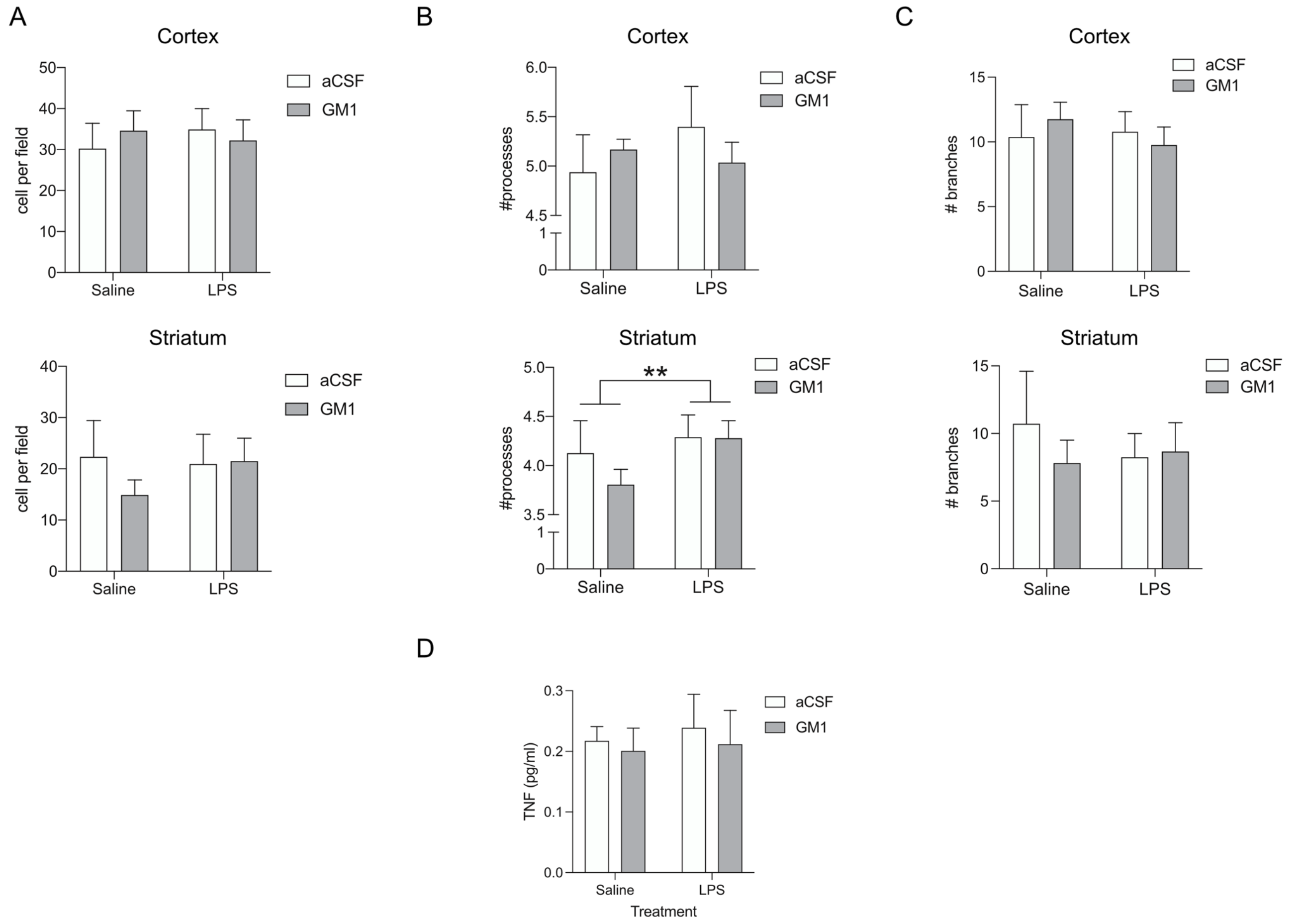

Additional Figure S4

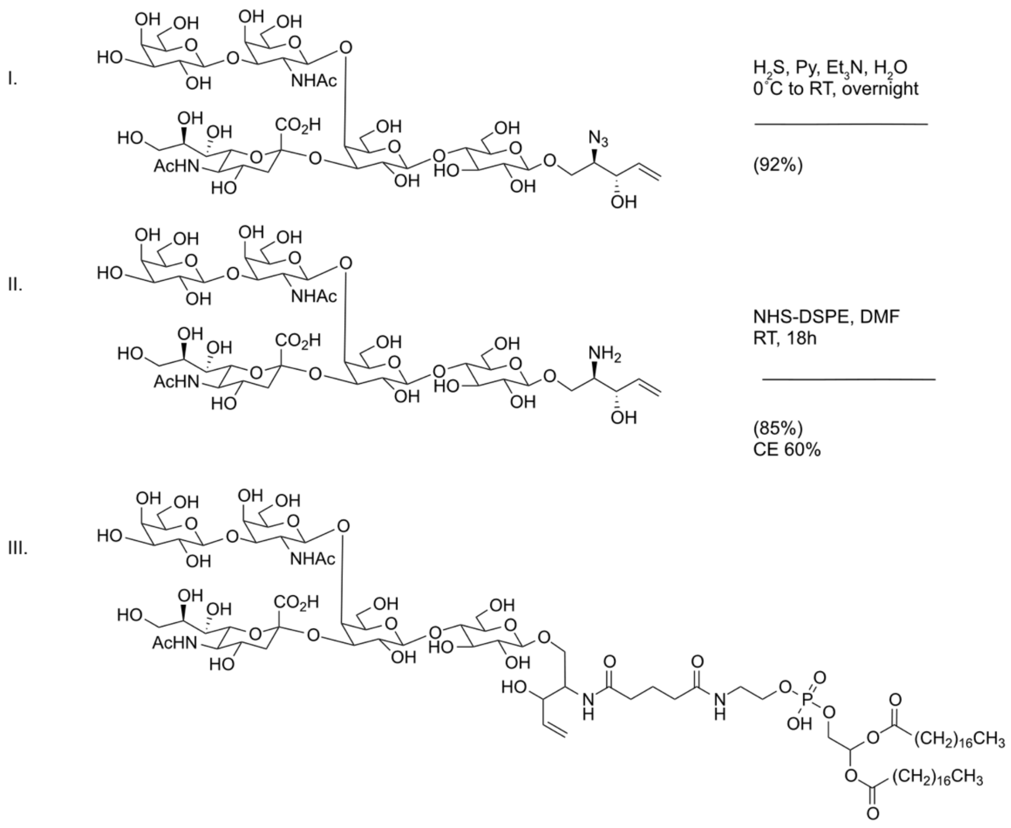

Additional Figure S5

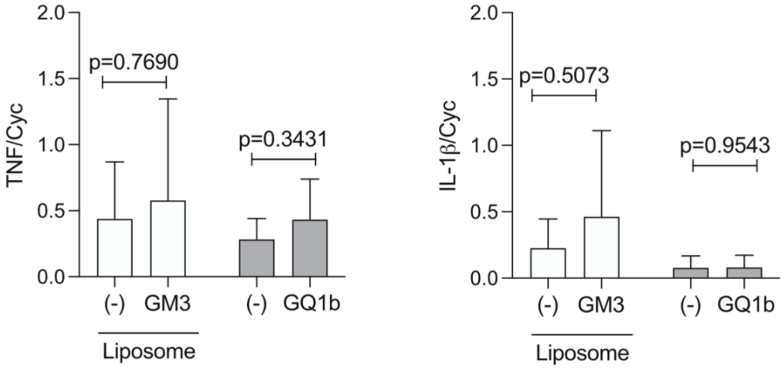

Additional Figure S6

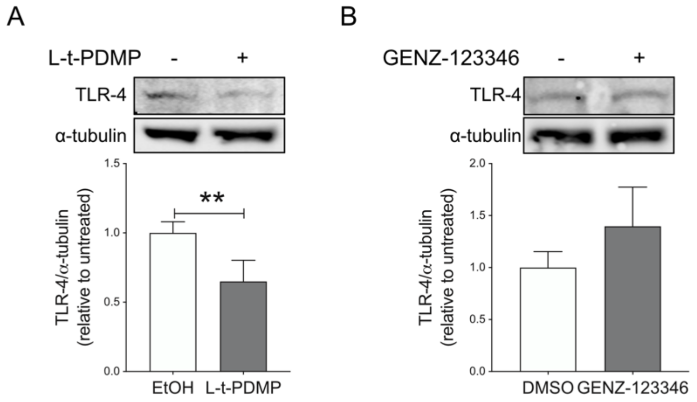

Additional Figure S7

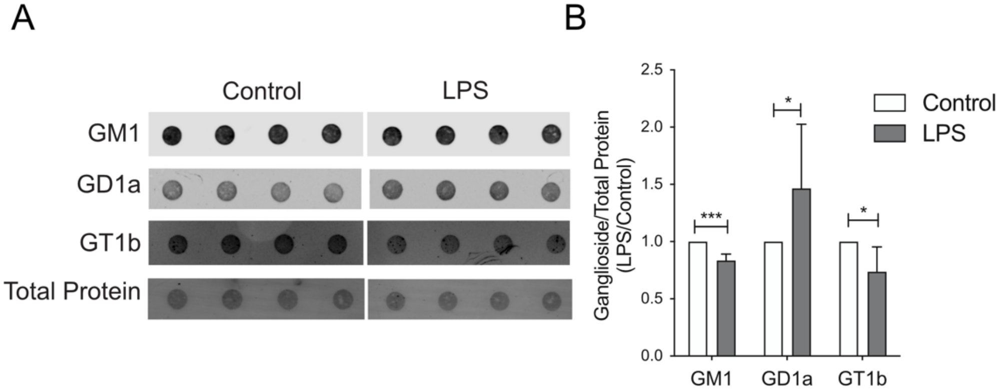

Supplement: Supplementary file 1 — Additional file 1. Additional methods for the analysis of cell viability and TLR-4 surface expression; additional figures S1-S7, including GM1 dose-response, cell survival after microglia incubation with exogenous GM1, TLR-4 protein expression, analysis of the effects of GM1 on Iba-1+ cell number and Iba-1 expression in vivo, synthesis and structure of GM1-DSPE, effects of GQ1b and GM3 on naive microglia, and cell ganglioside analysis after stimulation of microglia with LPS. [file 12974_2021_2374_MOESM1_ESM.pdf]
